# Laser Scanner-Based Hyperboloid Cooling Tower Geometry Inspection: Thickness and Deformation Mapping

**DOI:** 10.3390/s24186045

**Published:** 2024-09-18

**Authors:** Maria Makuch, Pelagia Gawronek, Bartosz Mitka

**Affiliations:** 1 Department of Land Surveying, University of Agriculture in Krakow, 31-120 Krakow, Poland; pelagia.gawronek@urk.edu.pl; 2 Department of Agricultural Land Surveying, Cadastre and Photogrammetry, University of Agriculture in Krakow, 31-120 Krakow, Poland; bartosz.mitka@urk.edu.pl

**Keywords:** terrestrial laser scanning (TLS), 3D technology measurement, 3D sensing, point cloud analysis, deformation measurement, multiscale model-to-model cloud comparison (M3C2)

## Abstract

Hyperboloid cooling towers are counted among the largest cast-in-place industrial structures. They are an essential element of cooling systems used in many power plants in service today. Their main structural component, a reinforced-concrete shell in the form of a one-sheet hyperboloid with bidirectional curvature continuity, makes them stand out against other towers and poses very high construction and service requirements. The safe service and adequate durability of the hyperboloid structure are guaranteed by the proper geometric parameters of the reinforced-concrete shell and monitoring of their condition over time. This article presents an original concept for employing terrestrial laser scanning to conduct an end-to-end assessment of the geometric condition of a hyperboloid cooling tower as required by industry standards. The novelty of the proposed solution lies in the use of measurements of the interior of the structure to determine the actual thickness of the hyperboloid shell, which is generally disregarded in geometric measurements of such objects. The proposal involves several strategies and procedures for a reliable verification of the structure’s verticality, the detection of signs of ovalisation of the shell, the estimation of the parameters of the structure’s theoretical model, and the analysis of the distribution of the thickness and geometric imperfections of the reinforced-concrete shell. The idea behind the method for determining the actual thickness of the shell (including its variation due to repairs and reinforcement operations), which is generally disregarded when measuring the geometry of such structures, is to estimate the distance between point clouds of the internal and external surfaces of the structure using the M3C2 algorithm principle. As a particularly dangerous geometric anomaly of hyperboloid cooling towers, shell ovalisation is detected with an innovative analysis of the bimodality of the frequency distribution of radial deviations in horizontal cross-sections. The concept of a complete assessment of the geometry of a hyperboloid cooling tower was devised and validated using three measurement series of a structure that has been continuously in service for fifty years. The results are consistent with data found in design and service documents. We identified a permanent tilt of the structure’s axis to the northeast and geometric imperfections of the hyperboloid shell from −0.125 m to +0.136 m. The results also demonstrated no advancing deformation of the hyperboloid shell over a two-year research period, which is vital for its further use.

## 1. Introduction

### 1.1. Problem Statement

Hyperboloid cooling towers for cooling process water by releasing thermal energy into the atmosphere are an essential component of the process line in many power plants today [1]. The working principle of natural-draught cooling towers is founded on the differential density of the air inside the tower and around it, with the specific weight of the former being reduced due to heating and an increased water vapour content. Air flows faster when the temperature difference between water and atmospheric air is higher [2,3]. One structural factor that directly affects the air flow rate in the tower, and thus its cooling performance, is the geometry of the sheet (also referred to as the shell) [4]. The specific geometry of the tower shell in the form of a one-sheet hyperboloid of revolution with bidirectional curvature continuity makes cooling towers stand out against other towers not only with regards to their different shape but also their several hundred times smaller ratio of the shell’s thickness to the structure’s size [5,6,7]. As these thin-shell structures are critical for the proper operation of the electrical power system, their operators strive to preserve their stability and durability, which hinge primarily on the condition of the reinforced-concrete shell [1,8]. The geometric parameters of hyperboloid structures and their changes over time are severe signs of cooling tower effort, especially important considering the ratio of its size to the shell thickness, defective construction, and poor repair practices regarding such objects [5,9]. Diagnosing the reinforced-concrete shell thoroughly is a complex task. It requires solutions adapted to specific construction conditions to ensure a comprehensive assessment of the structure’s geometry. The legal requirements to consider multiple factors of structural integrity prohibit simplifications commonly employed when investigating the geometry of such structures [10].

The sine qua non of a reliable interpretation of the cooling tower shell geometry is a sufficiently large and representative set of points on its surface. It should enable the engineer to determine radial deviations in relation to a one-sheet hyperboloid (as per the design or approximation) [11]. Geometric imperfections from continuous structural deformations, unrepaired construction defects, or improper reinforcement are important drivers of safety risks in thin-shell structures [12]. Still, engineers have to determine other geometric parameters as well that are often overlooked and yet determine the object’s suitability for continued service, such as deviation from plumb and the actual shell thickness (including its variability caused by repairs and reinforcement operations) [10,13,14]. The equation of the external surface of a cooling tower with its internal surface—defined in design documents with a quadratic equation or approximated with a theoretical model of a one-sheet hyperboloid—leads to erroneous interpretations of its geometric imperfections [15]. A thorough analysis of the geometric condition of a cooling tower requires that survey data be reduced to the internal surface (the proper reference surface) based on reliable information about the actual shell thickness distribution [4,16].

The specific shape of hyperboloid cooling towers, consequential for structural deformations and displacements, also determines the design, scope, and time of measurement. The primary challenge in reliably determining the geometry of such objects lies in the dependency of their deformations on factors variable in time, such as wind load, ambient temperature, or sun exposure [17]. To minimise their impact, the engineer must employ a rapid observation method to ensure as stable survey conditions as possible. Still, cooling tower failure reports suggest the need for more detailed measurements of hyperboloid structures targeting the shell’s geometry, particularly its local variability [5,18]. The application of terrestrial laser scanning (TLS) for this purpose, which offers datasets (point clouds) of millions of points and a fully metric, quasi-continuous registration of objects, can help overcome the discreteness of traditional surveying methods by expanding the scope of the monitored area over the entire structure [11,16,19,20,21,22]. The rapid pace of measurement with a laser scanner ensures constant observation conditions to minimise the impact of factors variable in time (which cause uncontrolled deformations and hinder the measuring accuracy of imperfections of the hyperboloid shell). Another noteworthy characteristic of TLS is that it does not interfere with the measured object. This way, the survey does not disturb the operation of the plant. One more important aspect is that scanning can occur in poor lighting or visibility and is entirely automatic [23,24,25].

The capabilities of terrestrial laser scanners offering remote and effective acquisition of reliable spatial data of high resolution have led to numerous attempts to apply them to deformation monitoring [26,27,28,29,30]. Their capability to detect structural deformations below the nominal accuracy of individual points in the cloud was demonstrated in the first attempts to employ terrestrial laser scanners to measure deformations [31]. Although terrestrial laser scanning offers large datasets critical to satisfactory results of structural geometry analyses [25,26,32,33], it requires a proper technique accommodating the specific local building practice to make the best use of the information contained in the point clouds.

This article presents an original concept for employing TLS for a complete assessment of the geometry of a hyperboloid cooling tower, taking into account the actual shell thickness distribution, which is often disregarded in tower geometry measurement designs. We verified several strategies and procedures for the reliable verification of structural verticality, the detection of signs of shell ovalisation, the estimation of the parameters of the structure’s theoretical model, and the analysis of the thickness and geometrical imperfection distribution in the reinforced-concrete shell (taking into account variations introduced by repairs and reinforcement) using three measurement series of a representative object, which was a cooling tower after fifty years of service.

### 1.2. Related Work

#### 1.2.1. Research Areas

The literature offers numerous case studies that confirm the effective application of TLS to monitor deformation and investigate the geometry of various objects. They also suggest (as evident from the large number of solutions employed) that the detection of millimetre-level structural deformations with terrestrial laser scanning remains a research problem to be addressed [24,25,34]. Considering the focus of the present research, the literature review was limited to publications on analysing structural geometry in two dimensions (useful for verticality verification and the determination of structural geometric imperfections), point cloud differentiation (applicable to shell thickness determination), and adjusting TLS datasets to a theoretical—design or approximate—structural model (useful in investigating geometric imperfections).

#### 1.2.2. Two-Dimensional Analysis of Structural Geometry

Examining an object’s geometry using two-dimensional TLS data involves highly detailed cross-sections extracted from point clouds. The consistency of the consecutive sections of the structure [35,36,37] and their geometric stability [38,39] can be analysed using juxtaposed cross-sections from the TLS data. Highly accurate centroids, dimensions, or cross-sectional areas (thanks to TLS data redundancy) facilitate a reliable analysis of deformation [40,41,42,43]. In the case of cooling towers, the analytical focus is on the coordinates of the circles’ origins (horizontal cross-sections) approximated from the point clouds. This approach was first employed by Schneider [44], who verified the verticality of a radio tower. Głowacki [45] also used centroids of cross-sections extracted from point clouds to calculate deviations from plumb of the centreline of a hyperboloid cooling tower. Then, he juxtaposed the inclination with the results of reflectorless measurements and confirmed the satisfactory accuracy of this approach. Other authors [46,47] investigated the verticality of cooling towers and plotted reliable plumb deviation graphs for thin-shell structures.

Cross-sections extracted from point clouds are also used to examine the geometric imperfections of towers. Teza and Pesci [41] used this method to verify the roundness of horizontal cross-sections of a heritage tower and generated a reliable map of its structural imperfections. Głowacki [45] determined the geometric imperfections of a wind turbine tower, an industrial stack, and a cooling tower based on the deviations of individual points in cross-sections from model circles. Kocierz et al. [48] effectively detected the millimetre-level deformations of a cooling tower shell by comparing vertical cross-sections extracted from a point cloud with their theoretical hyperboloid models.

In the present study, we used two-dimensional geometry analysis of cross-sections extracted from point clouds to assess the verticality of the hyperboloidal shell. We also used the horizontal cross-sections to analyse the geometric imperfections of the shell. However, in this case, we expanded a commonly used solution with an original method for detecting shell ovalisation, considering it a particularly dangerous anomaly in hyperboloid cooling tower geometry [49].

#### 1.2.3. Point Cloud Differentiation

The simplest method for comparing two sets of TLS data, the direct cloud-to-cloud (C2C) method, is used to resolve point cloud registrations based on the ICP algorithm [50,51]. It can calculate the Euclidean distances between two points of clouds to compare them. Every point of the first cloud is compared with the nearest point of the other cloud [52,53]). This approach to determining the shell thickness of a hyperboloid cooling tower has been employed recently by Zahradník [8]. The author directly estimated the distances between point clouds of the internal and external surfaces. However, the analysis covered only half of the structure, and the results were unsatisfactory because the measured shell thickness was less than the values in design documents [8]. The literature offers extensive discussions on the problem of the limited reliability of distances between TLS data computed using the C2C method, which is a sum of the actual distance, measurement noise, and systematic errors linked mainly to the registration, georeferencing, and stability of the reference frame [32,54,55,56,57,58]. Distance underestimation due to the juxtaposition of raw point clouds [59] calls for diverse methods for extracting or interpolating measurement data [60]. One commonly employed solution to improve the limited accuracy of a single point in a cloud is to interpolate the cloud into a regular grid [55]. This was what Van Gosliga et al. [61] carried out when they computed simulated deformations introduced onto the walls of an underground tunnel. Still, this method entailed a reduced TLS data resolution and required a special, local cylindrical coordinate system, restricting deformation detection to a single dimension [61]. A different approach, where the full TLS data resolution can be used so that geometric details of the object are available, involves comparing point clouds with a triangulated model of the feature (C2M (cloud-to-mesh)) [62,63]. Zogg and Ingensand [64] employed this approach to compare the as-designed viaduct TIN model with point clouds from consecutive measurement epochs. The authors then analysed the viaduct’s displacement and deformations during static loading. Still, as Barnhart and Crosby [63] noted, C2M requires detailed surface meshes to be generated, which entails complex computations and makes the method resource-intensive.

One solution that offers complete 3D point cloud comparisons, using TLS observation redundancy, a simple and high-performance alternative to reliable methods requiring detailed TIN models, is M3C2 (multiscale model-to-model cloud comparison) [53]. The M3C2 algorithm developed by Lague et al. [53], where the distances between two point clouds along locally determined normals to the investigated surfaces are computed, is typically used in geomonitoring [58,63,65,66,67]. M3C2-based solutions for differentiating point clouds while factoring in measurement uncertainty sources are also successfully employed in civil engineering diagnostics [57,68,69], comparing data from various measurement methods [70,71], and analysing the variability among point clouds acquired in different measurement conditions [72]. For instance, Law et al. [57] presented a periodic assessment of the surface degradation of a reinforced-concrete sea breakwater using distances between TLS data from consecutive measurements computed with M3C2. The results demonstrated the usability of the approach for identifying expanding defects and developing a structural renovation strategy, which is necessary for proper object management. M3C2’s capabilities regarding point cloud differentiation are confirmed by its various adaptations to specific circumstances [58,67,73]. The most common one, M3C2-EP, expands the estimation of the ‘level of detection’ (LoDetection, defined by Lague et al. [53]) with error propagation, which integrates internal measurement uncertainties and ununiform registration uncertainties to yield more reliable results for rough surfaces [67].

The solution for determining the reinforced-concrete shell thickness from TLS data is based on an estimation of the distance between point clouds of the internal and external surfaces of the structure. Considering the sources of uncertainty and limitations of C2C as potential reasons for the underestimation of the shell thickness [8], the distances between point clouds of the internal and external surfaces of the shell were determined using M3C2 [53]. When estimating the distances between TLS data, we considered the impact of measurement noise and the reliability of the orientation process, considering the local confidence interval for the computations.

#### 1.2.4. Comparison of TLS Data with Theoretical Model

Comparing point clouds with a design-based or approximate model of the feature involving distances from points to model surfaces is dedicated to objects of regular geometry [28]. The reference model can be defined based on design documentation or by approximating input TLS data. The literature has focused particularly on planes, cylinders, cones, parabolas, hyperboloids, or non-uniform B-splines [19,33,74,75,76]. This approach was confirmed effective by Van Gosliga et al. [61], who compared point clouds with a theoretical cylindrical model of an underground tunnel to analyse its deformations. Guidi et al. [77] also evaluated the actual shape of a parabolic trough collector by juxtaposing (noise-free) TLS data with a model geometry of the object. Comparing TLS data with a hyperboloid of revolution defined in design documents is a standard method for detecting geometric imperfections in cooling tower shells [4,9,15,21,45,78]. This approach is employed to analyse changes in the shape and deviations from the design of thin-shell structures introduced during their erection (construction defects) and the service of the cooling tower [15,46] and to confirm as-built parameters [4]. Suppose design documents are unavailable or there are significant discrepancies between the object and the design data. In that case, researchers also investigate differences between the point cloud (representing the actual geometry of the cooling tower) and a theoretical model of the hyperboloidal sheet approximated from TLS data [11,47,79]. Regrettably, these solutions commonly make use of measurements of the external surface of the cooling tower shell (which is not a model hyperboloid [15]) and disregard the variable thickness of the hyperboloid structure, which results in the erroneous interpretation of geometric imperfections. As mentioned by Bernardello and Borin [4], a thorough analysis of the geometric condition of a cooling tower requires that outer surface data be reduced to the internal surface (the proper reference surface as defined in design parameters or approximated with a theoretical model of a one-sheet hyperboloid) based on reliable information about the actual shell thickness distribution.

### 1.3. Research Significance

In TLS research on the geometry of hyperboloid cooling towers, the external surface of the shell tends to be treated as corresponding to its internal surface, which is defined by a theoretical model by design. This approach promotes erroneous interpretations of its geometric imperfections because a reliable analysis of the cooling tower’s geometric condition requires that data for the external surface be reduced to the internal surface (which is the proper reference surface) based on reliable information about the shell thickness distribution. This is a research gap concerning the application of TLS data in cooling tower geometry inspection. This article presents an original concept for employing terrestrial laser scanning to conduct a complete assessment of the geometric condition of a hyperboloid cooling tower as required per industry standards. The main innovation of this work is measuring the internal surface of the structure to determine the actual thickness of the hyperboloid surface. This information is used to estimate the parameters of the theoretical model and compute deviations in the hyperboloidal shell’s shape. Another relevant contribution is the effective, multi-stage registration of the point clouds of the structure’s interior and exterior using original reference targets and the rapid, innovative solution for detecting a particularly dangerous anomaly in the geometry of hyperboloid cooling towers, which is shell ovalisation.

## 2. Materials and Methods

### 2.1. Investigated Hyperboloid Cooling Tower

The proposed concept for verifying the geometry of a reinforced-concrete hyperboloid cooling tower was validated using an object in continuous service for over fifty years. The 9000 m^3^/h cooling tower was designed in 1959. The reinforced concrete shell of the structure is a hyperboloid of revolution with variable thickness. The target geometry of the shell that guarantees structural stability is defined through the following parameters:

- A height of 65.15 m;
- A height at throat level of 51.55 m;
- A radius at the level of the bottom ring of 21.52 m;
- A throat radius of 12.75 m;
- A radius at the level of the top ring of 13.52 m.

According to the design, the shell sits on 40 V-truss columns with a regular polygon cross-section. Their height in the shell plane is 2.85 m, and the base axis is on a 22.20 m circle. The design shell thickness near the columns is slightly larger and reaches 0.4 m, only to decline to 0.3 m at the top edge of the bottom ring. Above that, the shell thickness linearly changes to 0.12 m over a distance of 10.90 m. Then, it remains constant. The shell is topped with a reinforced-concrete stiffening ring that is 0.3 m wide at the wall face and projected 0.7 m outside.

### 2.2. Empirical Measurement Method

**Figure 1:**
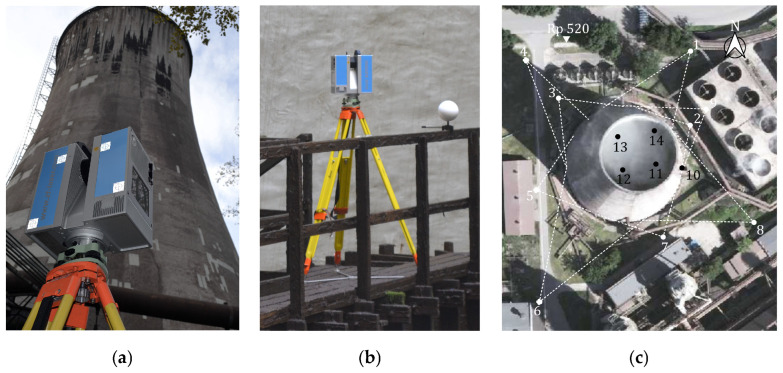
Terrestrial laser scanning of test cooling tower: (**a**) survey of external surface of shell with Z+F Imager 5010; (**b**) survey of internal surface of shell with Z+F Imager 5010; and (**c**) locations of scanning stations.

We scanned the test cooling tower with a Z+F Imager 5010 (Figure 1a,b) referenced to a stable, ten-point observation network with a reference frame tied to the local facility control network situated out of the process impact zone. The employed instrument, a Z+F Imager 5010, offers a maximum measurement range of 187.3 m, a spherical field of view (360° horizontally and 320° vertically), and a data acquisition rate of up to a million pixels a second. The angular accuracy of the device is 0.007° RMS, and its linearity error is below 1 mm at 50 m. The low level of noise facilitates the highest quality of data (the noise range at 50 m is 0.8 mm, 1.2 mm, and 2.7 mm for 80%, 37%, and 14% albedo, respectively). The instrument has a dual-axis compensator with a 0,007° accuracy and a laser plummet for a plummet accuracy of 0.5 mm/m. The IP53 protection class guarantees high dust and water tightness, which is a must in industrial settings. (The IP53 protection class protects against the ingress of particulate matter and liquids. It guarantees the isolation of dust and spray of water from any angle from 60° to vertical on all sides).

**Figure 2:**
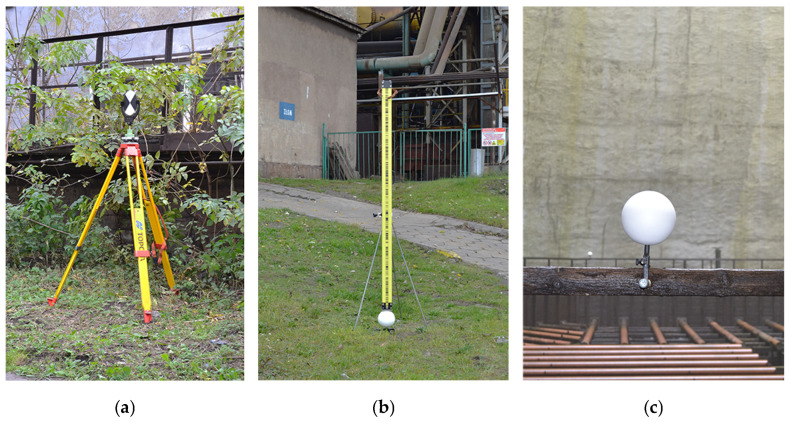
Reference objects: (**a**) targets of Z+F Professional; (**b**) steel reference sphere during precise levelling; and (**c**) steel reference sphere fixed to the railing.

The external shell was scanned three times from nine stations (1–9) around the structure (Figure 1c). The stations were selected to optimise TLS data acquisition for the entire shell, factoring in the industrial profile of the site, technical parameters of the scanner, and limited measurement time. Each measurement series followed predefined organisational constraints to minimise the influence of external factors on the measurement results of the hyperboloid structure. The limited observation duration was considered a guarantee for the static model of the structure. The particular care for uniform and constant measurement conditions reduced the impact of outside influences. The measurements were taken in the early hours of the day, on cool days with an overcast sky and windless weather. The method we employed, whereby the scanner was put at stations of known coordinates and tied to well-defined references in the point cloud, guaranteed the registration of nine point clouds from each session and proper georeferencing in the reference frame. Thanks to the compatibility of the Z+F Imager 5010 and the B&W Z+F Professional rotary tilting 6″ targets (Figure 2a) with high-precision Leica GZR3 carriers (centred and levelled over the observation network points), we were able to use the multi-station method to complete the observations.

The planar positions of the scanner and targets were assumed per the horizontal coordinates of the observation network points. Their heights were measured through trigonometric levelling, factoring in laboratory-determined offsets: reflector–target–scanner. Additionally, to control the constant height of the scanner and targets, we employed original specially made 150 mm steel spheres with adapters to ensure they sat stably in the field (Figure 2b). The spheres were distributed evenly around the cooling tower and precisely levelled with Leica NA3003 by placing Leica GPCL2 invar bar code staffs directly on the reference spheres (Figure 2b). Each measurement was referenced to the benchmark Rp 520 (Figure 1c) (the tie-in point for all research projects), assuming a 0.2 mm tolerance for double measurement. The heights of the centres of the spheres in the frame of reference were identical to the centroids of the sphere models, thus ensuring height control for the TLS data. The reference spheres were also used as points for tying adjacent point clouds to improve registration.

We surveyed the interior of the structure in addition to the external surface to compute the actual shell thickness (Figure 1b). We needed to find a way to tie interior measurements to the observation network outside, which realises the frame of reference, to ensure consistency and coregistration. To this end, we measured the coordinates of point 10 positioned on a tripod at the entrance to the structure (the reference for the internal survey) and signalled with a Leica GPR121 PR0 circular prism from three points of the observation network using Leica TC 2003. The intersected point with known coordinates was first used as a scanning station tied to reference objects outside and inside the structure. Next, we installed a Z+F Professional target at the point at the entrance to the structure (Figure 2a) as a tie-in for measurements inside the structure from four stations (11–14) distributed at regular intervals around a wooden platform (Figure 1c). Considering the poor stability of the wooden structure, we used reference objects that did not require rotation: 150 mm steel spheres fixed to the railing of the platform with original adapters designed and produced specifically for this purpose to ensure stable and permanent installation for the time of the survey (Figure 2c).

### 2.3. Point Cloud Registration and Georeferencing

This project required that the TLS data be registered and georeferenced by aligning the point clouds into a stable frame of reference for each measurement series. These steps are critical for high-precision engineering jobs because they are decisive for the reliability of the final results [60]. The propagation of registration and georeferencing errors is the primary source of uncertainty (systematic errors) that hinders the analytical reliability of TLS data [25]. This is why we defined the registration and georeferencing methods already when designing the surveys. The positions of the scanner, targets, and reference spheres were consequential for acquiring point clouds in the frame of reference. Furthermore, the data could be precisely aligned only with the minimum (adjacent) scan overlap of 30%. The registration and georeferencing followed a hybrid regime, combining the following:

- The direct method when the position and tie link of the scanner were known. It is the optimum, most time-effective, and efficient method. Still, its accuracy hinges directly on the quality of the scanner’s positioning and orientation [80,81].
- The indirect method based on artificial reference points (well-defined targets or reference spheres in point clouds). It is the most common method in engineering due to its reliability and accuracy, but the latter depends on the even distribution and fixed position of tie points [82].
- The cloud-to-cloud method based on the IPC (iterative closest point) algorithm [50]. Each iteration reduces the distance between two point clouds until the minimum value is reached. Still, it has to be initiated with a pre-registration, requires at least 30% overlap, and data free of objects changing over the measurement time (such as trees) [83].

We divided the shell TLS data registration and georeferencing process with the three methods into several stages. The procedure—identical for all three series—was realised in Cyclone. First, we estimated the transformation parameters using the direct method. The point clouds were aligned into the frame of reference based on the known positions of the scanner and their links to the targets. The centres of the B&W targets were extracted automatically thanks to the high contrast of the reflected beam. Then, we (indirectly) registered the point clouds using the targets. The registration was reinforced with ten reference spheres regularly positioned around the tower, significantly improving the survey’s geometry. We determined the centroids of the reference spheres automatically, using sphere models generated with the least-squares method.

The tie-ins of the point clouds based on the reference points (targets and spheres) on the ground and at eye level covered a limited portion of the scene only. Seeking to improve the orientation quality in areas without identifiable points and to improve the geometry, we resorted to cloud-to-cloud registration (assuming a statical model of the structure), which searches for the best match of data using the ICP algorithm [83]. Thanks to the automatic creation of links between the point clouds based on on-the-fly registration and georeferencing, we did not have to manually pinpoint homologic points in the scans, and the frame of reference was preserved. We streamlined the computations by manual data filtration to remove moving objects and noise. We prevented error propagation using the ‘closed loop’ principle, whereby every model point cloud was a dataset for the subsequent adjustment, and the first and last scans were adjusted.

The first step towards registering the TLS data for the interior was registering the point clouds from four stations inside the tower and one station at the entrance. This stage was based on six reference spheres fixed to the platform railing with original adapters (this way, no target rotation was necessary, which we considered a potential source of errors as the wooden structure was unstable) and the target at the entrance. Ties based on the reference spheres (fixed to the wooden railing) covered a limited part of the scene. We improved the orientation quality in areas without well-defined targets with automatic ties between point clouds using the ICP algorithm (cloud-to-cloud registration). In the next step, we linked the outcomes of the interior data registration with oriented scans of the external surface to obtain proper georeferencing (nested registration). The registered internal and external point clouds were tied through three targets, three reference spheres, and the known position of the station at the entrance. 

### 2.4. Deviation from Plumb: Verification of Roundness of Cross-Sections

The operational safety of thin-shell structures can be reliably measured by determining any changes in the position of the cooling tower foundations relative to their initial position, which is directly qualitatively and quantitatively connected with any deviation of the structure’s axis from plumb and the ovalisation of the reinforced-concrete shell [49]. The deviation from plumb has to be considered already when estimating the parameters of the structure’s theoretical model and in static strength calculations [6]. We verified the deviation from plumb using the TLS data and an algorithm for the two-dimensional analysis of point data, which has been successfully employed for tower structures [41,44,46]. We determined the structure’s deviation from plumb by fitting circles of horizontal cross-sections into selected data, observing the condition of the minimisation of a sum of squared deviations v_i_. We determined the deviation of the axis from plumb by analysing the shifts in the origins of the circles extracted from point cloud sections on consecutive levels (cross-sections of observations) at an accuracy greater than the accuracy of a single point acquired with the scanner thanks to data redundancy [44].

**Figure 3:**
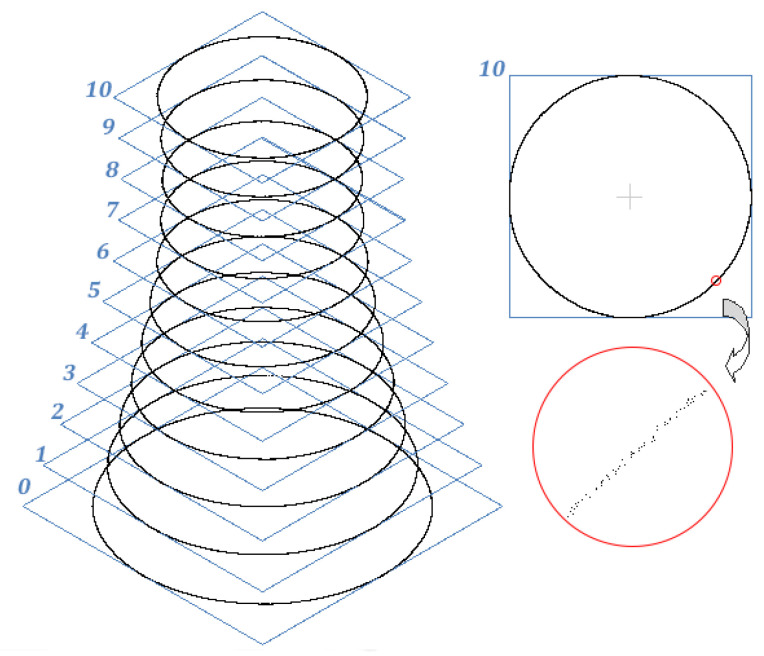
Horizontal cross-sections for verticality verification.

The problem was solved by estimating coordinates $X_{s_{i}}$, $Y_{s_{i}}$ of the origins of the horizontal cross-sections and components of axial deviation from plumb ($w_{x_{i}},w_{y_{i}}$). The solution was to determine the parameters of approximate circles based on points on each level with the least-squares method. We took eleven 5-millimetre, manually extracted sections of a point cloud projected onto a plane of each level (Figure 3) to ensure a sufficient number of observations defining the structure’s sections. The heights of the levels (at 6.5 m intervals) were selected to facilitate identifying alarming signs of structural deformations (lateral drift and ovalisation) while minimising the analytical effort. The unknown parameters of the *i*-th level were the coordinates $X_{s_{i}}$, $Y_{s_{i}}$ of the origin and the radius $R_{i}$. Our calculations based on the planar coordinates of the point cloud sections employed a parametric circle equation:

(1) $$x^{2}+y^{2}+xA+yB+C=0$$

(2) $$A=-2X_{s_{i}}\;B=-2Y_{s_{i}}\;C=X_{s_{i}}^{2}+Y_{s_{i}}^{2}-R_{i}^{2}$$

The estimated parameters of the *i*-th level are described with the following equations:

(3) $$X_{s_{i}}=-\frac{A}{2},\;Y_{s_{i}}=-\frac{B}{2},\;R_{i}=\sqrt{X_{s_{i}}^{2}+Y_{s_{i}}^{2}-C}$$

The multipoint cross-sections provided redundant observations. The average number of points on a single level was 25,070, which ensured reliable information about their geometry. We fitted circles into selected points of each of the eleven levels using the method for the minimisation of a sum of squared deviations, estimating parameters A, B, and C as unknowns to determine values $X_{s_{i}}$, $Y_{s_{i}}$ and the fit accuracy. We then used the estimated coordinates of the origins of the *i*-th cross-sections to compute the components of the deviation of the structure’s axis of revolution from plumb at the *i*-th level ($w_{x_{i}},w_{y_{i}}$) compared with the zero level (reference) from:

(4) $$w_{x_{i}}=X_{s_{i}}-X_{s_{0}},\;\;w_{y_{i}}=Y_{s_{i}}-Y_{s_{0}}$$

Next, we considered the distribution of geometric imperfections of the structure based on the cross-sections extracted from the point clouds [41]. We calculated millimetre-level roundness deviations for each point of the *i*-th cross-section as non-conformities of the actual shell geometry with the approximated model circle. To detect deformations in the cross-sections, especially ovalisation, which is a particular stability hazard, we generated a hypsometric visualisation of the identified discrepancies in CloudCompare and performed an approximate assessment of the fit of empirical distributions to theoretical normal distributions. By associating specific values of millimetre-scale roundness deviations with particular points in the cross-sections and colour visualisation (with identification of the scalar field (SF)), we could perform a detailed analysis of the geometric imperfections and the detection of shell ovalisation signs as hazardous anomalies in the geometry of the hyperboloid cooling tower [49]. We then verified the consistency of the frequency distributions of the identified discrepancies with the expected theoretical distribution using histograms and density curves of normal distributions. Visualising the frequency distribution of the millimetre-level roundness deviations with bar graphs allowed us to verify their aspects of bimodality, which we used as an original detector of shell ovalisation.

### 2.5. Shell Thickness Map: Reduction of Measurements to the Internal Surface

A thorough analysis of the geometric condition of a cooling tower requires that survey data be reduced to the internal surface (the proper reference surface) based on reliable information about the actual shell thickness distribution [16]. Thin-shell structural thickness data are also necessary for verifying the load-bearing capacity and service limit states. Structural condition analyses should cover the designed variable shell thickness distribution (due to internal forces and stresses within the thin shell) and its variations caused by construction errors, service damage, and repairs and reinforcements of the shell [6]. The cooling tower shell thickness distribution information comes from structural parameters defined in the design, as-is, and service documentation. Sadly, data in company archives are often fragmented, incomplete, and even wrong. Therefore, the shell thickness distribution has to be approximated based on cylindrical cores drilled along meridians of thin-walled structures. The inconvenience of core drilling and the hazardous consequences of the destructive testing method, which harms the load-bearing capacity of thin-shell structures, limit drilling possibilities. The shell thickness distribution has to be determined using successive approximations, which is not very reliable [13].

The solution for determining the reinforced-concrete shell thickness from TLS data is based on estimating the distance between point clouds of the internal and external surfaces of the structure. A special approach had to be employed to differentiate TLS datasets at an accuracy exceeding the nominal accuracy of individual points in the clouds. The primary assumption was to make good use of the high redundancy of the TLS data [32] while considering the limited computational capabilities. In addition, we used an algorithmic toolset that guarantees the independence of the proposed solution of data density variance and can determine the shell thickness in a three-dimensional space. Considering the sources of uncertainty and the limitations of the cloud-to-cloud method as potential reasons for the underestimation of the shell thickness, the distances between point clouds of the internal and external surfaces of the shell were computed using the M3C2 algorithm [53]. The M3C2 method exhibits greater accuracy (the theoretical accuracy of the parameters of the model estimated from point clouds significantly exceeds the point accuracy of the scanner [26,62]) thanks to making use of TLS data redundancy. It is a simple and efficient alternative to reliable methods where detailed TIN models must be constructed. Additionally, M3C2 takes into account noise impact and orientation reliability when estimating distances in TLS datasets [63].

The distance between the TLS datasets for the external and internal surfaces of the cooling tower was computed in CloudCompare with the M3C2 distance plugin. First, M3C2 determined the direction of internal and external cloud differentiation from vectors normal to the analysed surfaces as the average direction of the normals for both datasets. Normals that defined the data orientation (determined considering the local geometry) were estimated for planes of the best fit to the nearest neighbours defined with a radius of d/2 as a parameter of the algorithm (projection scale). M3C2 has a limitation: an assumption of the local flatness of the compared surfaces [58]. We overcame it by assuming the value of parameter d, which determines the directions of normal vectors as a function of the surface curvature, local point cloud density, and analytical level of detail, to be 0.1 m. By generating a cylinder of radius 0.05 m and an axis consistent with the selected direction of data differentiation, the algorithm defined a coordinate system inside which the resultant distances between the two point clouds were determined (as the spatial averaging of all distances computed inside the cylinder). We limited the data differentiation range by defining the maximum cylinder height (0.65 m) to streamline the computations while encompassing the entire shell thickness.

We then combined the cloud-to-cloud distances with direct shell thickness measurements at the interface with the columns to determine the variable distribution of the shell’s thickness. The M3C2 algorithm assigned a specific value (scalar field) to every point of the external surface cloud (the result set) so that the results could be visualised in three dimensions. Considering the popularity of planar representations, where the condition of a hyperboloidal shell can be presented clearly in two dimensions [79], we present the results also on a two-dimensional map generated in CloudCompare with the qSRA plugin (surface of revolution analysis). The tower shell surface (hyperboloid) was non-developable, so it had to be mapped onto a developable surface to be presented on a plane. The shell thickness map was a metric projection of scalar field points onto the side of a cylinder with the base identical to the lower edge of the tower shell developed along it onto a plane.

We used the identified parameters of zones of hyperboloidal shell thickness distribution, which define the function of the thickness of the reinforced-concrete wall in height, to reduce the coordinates of points representing the external surface to their counterpart points of the internal surface (taking into account the thickness of the reinforcing layer). To reduce the computation effort, the procedure followed a reduction in excessive data that would hinder effective processing. We reduced the point cloud sizes using an octree structure, which offers a recurrent division of a 3D scene into smaller regular fragments [84]. The size of the TLS data was reduced in CloudCompare by interpolating the point clouds into a regular distribution, reflecting the data division level defined by the sizes of the resultant sets of 200 million points. The algorithm assigned each point in the clouds to a cube based on its location and determined the centroid of every cube based on the points to increase (thanks to TLS observation redundancy) the limited accuracy of a single point. The set of centroids instead of the initial point cloud resolved the data size problem and improved the accuracy of individual points (based on averaged observations). The three series of TLS data were interpolated into regular distributions and transformed into a cylindrical coordinate system (linked to the direction of the primary axis of the structure estimated from horizontal cross-sections and the eastward *x*-axis) and reduced to the internal surface through radial corrections as required for each level individually.

### 2.6. Estimation of a Theoretical Model and Geometric Imperfection Analysis

The repair of hyperboloid shells is estimated primarily by determining deviations in their geometry by comparing a set of observations for the internal surface with the model hyperboloid (designed or approximated). When design documents with parameters for a theoretical model are unavailable or insufficient, the values have to be estimated. The internal surface is a hyperboloid of one sheet, a quadric surface that satisfies the following general quadric equation [85]:

(5) $$F\left(x,y,z\right)=a_{11}x^{2}+a_{22}y^{2}+a_{33}z^{2}+2a_{12}xy+2a_{13}xz+2a_{23}yz+2a_{14}x+2a_{24}y+2a_{34}z+a_{44}$$

The specific form of the shell can be approximated after including appropriate assumptions in the standard equation (a_11_ = a_22_ and a_12_ = 0) that bind the estimated coefficients a_ij_ determining the parameters that describe the position and shape of the model hyperboloid [11].

To estimate the dimensions of semi-major axes of the approximated surface that define the details of the theoretical model’s shape, one has to transform function *F*(*x*,*y*,*z*) into a standard form without cross terms and with the minimal number of linear terms. The problem boils down to the transformation of coordinates: the rotation of the system’s axis according to the major axes and the translation of the system origin to the centre of the quadric (by calculating the roots of the characteristic polynomial). In a Cartesian coordinate system consistent with the directions of the surface’s axes of symmetry, the theoretical model of a one-sheet hyperboloid, where parameters *a* and *b* are the dimensions of the semi-transverse axes of the hyperboloid and parameter c defines the dimension of the semi-conjugate axis, is described with the following equation [85]:

(6) $$\frac{x^{2}}{a^{2}}+\frac{y^{2}}{b^{2}}-\frac{z^{2}}{c^{2}}=1$$

The verification of the roundness of the horizontal cross-sections of the structure (indicative of minor deviations in the cloud point from the theoretical model) and the transformation of the coordinates into a system consistent with the major axes of the quadric (considering the identified deviation of the axis of rotation from plumb), and with the system origin in the centre of the structure’s base, paved the way for assuming a theoretical model of the object as a surface described with the equation for a one-sheet hyperboloid of rotation built by rotating a hyperbole (parameters *a* and *c*) about the conjugate axis of symmetry (*a* = *b*) translated along the OZ-axis (by z_o_) in the form of:

(7) $$\frac{x^{2}+y^{2}}{a^{2}}-\frac{{\left(z-z_{o}\right)}^{2}}{c^{2}}=1$$

Based on the assumptions and dependency first made, we estimated the theoretical model parameters by extending the simplified two-dimensional approach (excluding the estimation of the general quadric equation), which involved fitting branches of the hyperbola into measurement points describing the generator of the cooling tower shell on one of its vertical cross-sections (employing the criterion of minimisation of a sum of squared deviations [16]) to the approximation of a one-sheet hyperboloid of revolution in a three-dimensional space. The implementation of this involved the computation of three parameters of the theoretical model of the structure, *a*, *c*, and *z_o_*, with the least-squares method based on observations reduced to the internal surface (the size of which was restricted to 200 thousand points) in a cylindrical coordinate system consistent with the primary axes of the quadric (where R = √(x^2^ + y^2^)) using the function of radius vs. height *R*(*z*)*:*

(8) $$\left(z\right)=a\sqrt{1+\frac{{\left(z-z_{o}\right)}^{2}}{c^{2}}}$$

We identified the geometric imperfections in the reinforced-concrete structure defined as the radial distances from the TLS observations (interpolated to a regular distribution and reduced to the internal surface) and the theoretical sheet with the model of a one-sheet hyperboloid of rotation described with function *R*(*z*), which was the mean of the estimated parameters. The point clouds from consecutive series were compared with the theoretical model (defined by the heights and radii assigned to them) in CloudCompare with the qSRA plugin. The identified radial deformations (differences between the measured and theoretical radii) were assigned to each point in the cloud as a specific SF value for the hypsometric visualisation of the results. The geometric imperfections in the reinforced-concrete structure were presented in a three-dimensional space (point clouds) and as a two-dimensional map, which is a metric projection of the scalar-field points onto a side of a cylinder with the base identical to the lower edge of the tower shell developed along it onto a plane.

## 3. Results and Discussion

### 3.1. TLS Data Registration and Georeferencing

The TLS data were registered and georeferenced using a hybrid method, amplifying flaws in the geometry. It combined a direct and indirect method based on reference objects and the ICP algorithm. The synergistic approach to the registration and georeferencing allowed us to use their advantages and eliminate their drawbacks. It was also an attempt to make the process as automated as possible. The general accuracy of the outcomes of the multi-stage registration and georeferencing process was described with the RMS. Defined as the average orientation error and reflecting the quality of all links used in the orientation, the RMS did not exceed 2 mm. The accuracy of the transformation of the point cloud coordinates from the reference frame (for a specific measurement session) into the global coordinate system was verified by reading the coordinates of the observation network points from the clouds. The mean data consistency was ±1 mm. The maximum divergence was ±2 mm horizontally and ±2 mm vertically.

**Figure 4:**
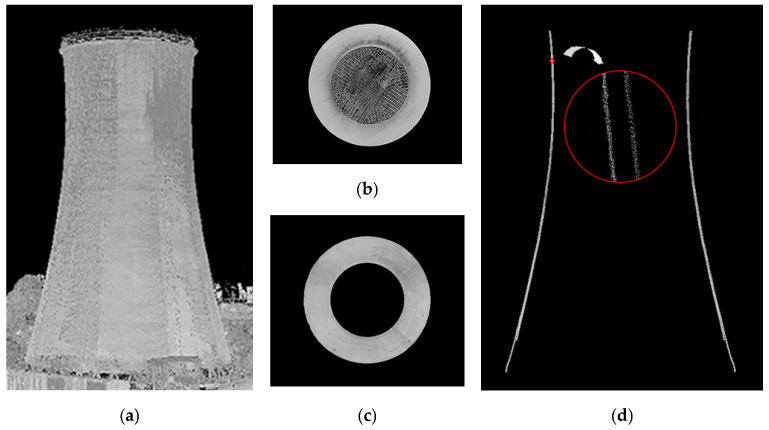
Results of point cloud registration and georeferencing—series III: (**a**) external point cloud; (**b**) internal point cloud; (**c**) external and internal point cloud after filtration; and (**d**) vertical cross-section of the external and internal point cloud.

The quality assessment and independent verification of the registration and georeferencing processes were ensured by the reference spheres with original adaptors that ensured their fixed and stable positioning on the ground. The heights of the ten spheres positioned at constant intervals around the object were determined through the rigorous adjustment of elevations measured with a precision level tied to benchmark Rp. 520. The adjusted heights of the reference spheres (factoring in their radii) determined the heights of their centroids, which were the quality control points, down to 0.0003 m. The registration and georeferencing of the external surface were verified by comparing the heights of the centroids of the spheres from the oriented TLS data ($H_{TLS}$) with those determined through the strict adjustment of precision levelling heights ($H_{NIW}$). The discrepancies ($\Delta H=H_{TLS}-H_{NIW}$) did not exceed 0.002 m for the three measurement sessions, which seemed to validate the proposed multi-stage hybrid pipeline for point cloud registration and georeferencing (Figure 4a,b).

The reliability of the resulting nested registration was demonstrated through analysis of the coaxiality of the internal and external point clouds (Figure 4c). The mean discrepancies between coordinates $\Delta X_{s_{i}}$, $\Delta Y_{s_{i}}$ of the origins of the ten circles estimated as per the procedure presented in Section 2.4. was ±1 mm. The maximum divergence was ±2 mm on the *x*-axis and ±3 mm on the *y*-axis.

The visual consistency of the resultant data was an indispensable confirmation of the correct orientation of the point clouds (Figure 4). The shape of the test object facilitated a rapid verification of the coherence of the observation results using vertical and horizontal cross-sections of the structure. Point clouds with at least 30% overlap filtered manually allowed us to determine the coherence and continuity of the data, confirming a successful process (Figure 4d).

### 3.2. Deviation from Plumb: Verification of Roundness of Cross-Sections

**Figure 5:**
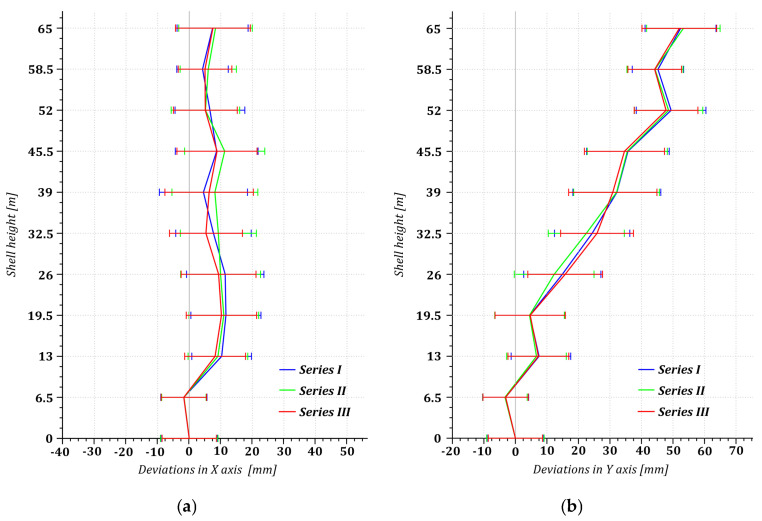
Plots of deviations from plumb: (**a**) on the ZX plane; (**b**) on the ZY plane.

The deviation of the structure’s axis from plumb was determined by the shifts in the origins of the circles extracted from point cloud sections on ten levels. The components $w_{x_{i}},{\;w}_{y_{i}}$ of the structure’s lateral drift determined by the shifts in the origins of the *i*-th cross-section compared with the origin of the zero cross-section are presented as lateral drift plots in the ZX plane and ZY plane, taking into account the accuracy of fitting (with the standard deviation represented as an error bar in millimetres [45]) (Figure 5).

The verticality analyses demonstrated a permanent and constant (over the two-year research cycle) lateral drift to the northeast, increasing with height, indicating a change in the initial location of the cast-in situ foundation of the cooling tower. We were unable to confront our findings with observations of the vertical displacement of the control benchmarks (stabilised in the foundation during construction), the purpose of which was to identify the extent and direction of the foundation and top tilting because of their instability (six out of eight were damaged) and incomplete data in company archives. The missing documents on measurements of changes in the initial positions of the benchmarks and the lack of as-built reports prevented us from identifying the nature of the lateral drift of the hyperboloidal cooling tower (due to construction errors or service).

**Figure 6:**
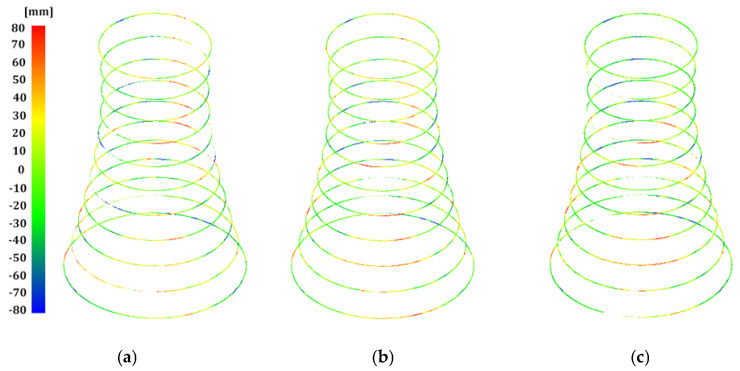
Roundness deviations in horizontal cross-sections: (**a**) series I; (**b**) series II; (**c**) series III.

**Figure 7:**
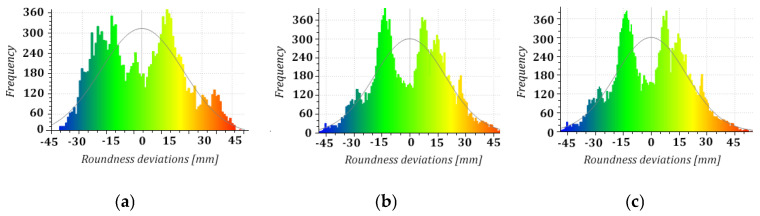
Distribution of roundness deviations in horizontal cross-section 9: (**a**) series I; (**b**) series II; (**c**) series III.

**Figure 8:**
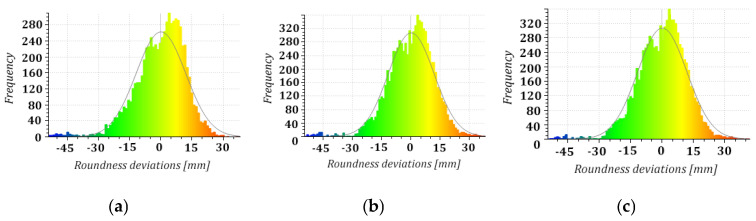
Distribution of roundness deviations in horizontal cross-section 1: (**a**) series I; (**b**) series II; (**c**) series III.

Next, we considered the distribution of the geometric imperfections of the structure based on the cross-sections extracted from the point clouds. Roundness deviations, being non-conformities of the actual shape of the shell with the approximated model circle for individual points in the cross-section and represented with colour visualisations of the scalar fields, provided consistent results for all measurement series (Figure 6). Additionally, we proposed bar graphs to represent the frequency distribution of millimetre-level roundness deviations to detect ovalisation, which threatens the structure’s stability [49]. Our analyses covered the normality of divergency distributions as a criterion of their randomness and the bimodality of their distribution as an original detector of shell ovalisation. They demonstrated a permanent (considering the two-year study period) elongation of the diameter of the cross-sections along the axis of the structure’s deformation (lateral drift). There were alarming signs of shell ovalisation on 5 levels (5–9) detected with the bimodal distribution of divergences (Figure 7). We excluded a backward ovalisation of the structure’s head (crosswise to the lateral drift axis) typical of imminent failure conditions (Figure 8) resulting from excessive efforts in the reinforced-concrete shell due to advancing deviation from plumb.

### 3.3. Cooling Tower Shell Thickness

**Figure 9:**
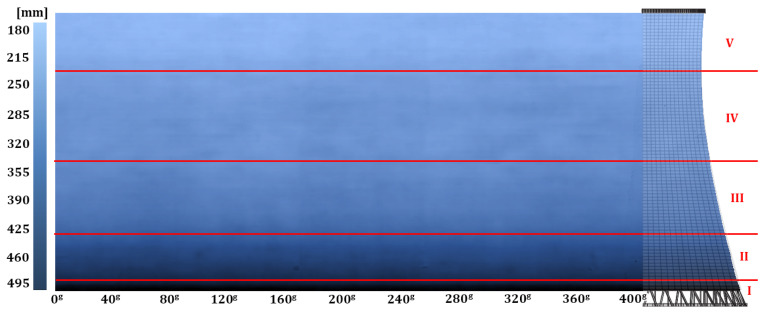
Cooling tower shell thickness map with zones of varied thickness distribution. Horizontal: shell development azimuth; vertical: shell height.

The reinforced-concrete shell thickness was determined from the TLS data based on an estimation of the distance between point clouds of the internal and external surfaces of the structure. The TLS datasets could be differentiated with an accuracy exceeding the nominal accuracy of individual points in the cloud and factoring in sources of uncertainties thanks to the M3C2 algorithm. To ensure the reliability of the approach, it was necessary to keep the differentiated point clouds coherent, as demonstrated in the analysis of the divergences identified in coordinates $\Delta X_{s_{i}}$, $\;\Delta Y_{s_{i}}$ ($\sum \Delta X_{s_{i}}=0.0017\;m,\;\sum \Delta Y_{s_{i}}=0.0005\;m$) of the origins of the ten circles estimated from sections of the oriented TLS data. The results could be visualised in three dimensions because every point of the external surface cloud (the result set) was assigned a specific value of shell thickness (scalar field). Considering the popularity of planar representations of the condition of a hyperboloidal shell in construction practice, we also present the results in a two-dimensional map. The shell thickness map is a metric projection of scalar field points onto the side of a cylinder with the base identical to the lower edge of the tower shell developed along it onto a plane (Figure 9).

The map of the cooling tower shell thickness (Figure 9) identified five zones (I, II, III, IV, and V) where the differentiation of the thickness distribution vs. height was defined by various functions. The thickness was the greatest at the interface with the columns (0.495 m). It then dropped to 0.395 m at the top edge of the bottom ring (zone I). In zone II, the thickness declined to 0.215 m, which was the dominant value in the central part of the structure (zone III), and then was further reduced to a constant value of 0.198 m (zone IV). In the last zone (V), from the throat to the top stiffening ring, the reinforced-concrete shell was the thinnest (0.182 m). Each zone exhibited a local variability in shell thickness (±0.01 m) due to repairs and reinforcing operations. The shell thickness for individual parallels was determined based on average values.

We considered the resultant distribution of the shell thickness conforming to design documents and overhaul documents from 1990 to 1991. The repair recommendations from that time involved the reinforcement of the structure by increasing its thickness on the external surface (from the columns to the top ring) with a layer of reinforcing steel mesh of a variable bar diameter with an overlay of shotcrete. The shell thickness map seems to confirm the reinforcement treatment (the state after the 1990–1991 overhaul has never been confirmed so far). By juxtaposing the measured parameters of the shell with the design documents, we identified the variable thickness of the reinforcing overlay. In the upper zone, above the throat, it was 62 mm (4 mm bars). In the middle part (zone IV), the overlay thickness was 78 mm (6 mm bars). The reinforcing layer in zones I, II, and III was 95 mm with a bar diameter of 8 mm.

### 3.4. Estimation of Theoretical Parameters and Geometric Imperfection Analysis

The parameters of the structure’s theoretical model were estimated using a simplified two-dimensional approach [16] extended to three-dimensional space. The implementation of this solution facilitated the computation of three parameters of the theoretical model of the structure (a, *c*, and *z_o_*) with the least-squares method based on observations from consecutive measurement series reduced to the internal surface and computation of its accuracy of fitness to the cloud point set (σ_o_), reflecting any non-conformity of points representing the internal surface with the equation of the approximated surface (Table 1).

The dimensions of the semi-transverse axis (*a*) and semi-conjugate axis (*c*) and the structure’s height at the throat ($z_{o}$) calculated through a system of approximating equations for observations from the three measurement series demonstrated minuscule variability (Table 1). With function *R*(*z*), which describes the relationship between the radius and structure’s height, we determined, for each measurement series, the values of the radii at the level of the bottom ring (*z* = 0), at the throat (*z =* $z_{o}$), and at the top ring (*z = z_max_*), which we then compared with the data in the design documents (Table 2). The conformity of the results seemed to confirm the reliability of our approach for estimating the parameters of a hyperboloid cooling tower theoretical model, necessary when design documents are unavailable or unreliable.

**Figure 10:**
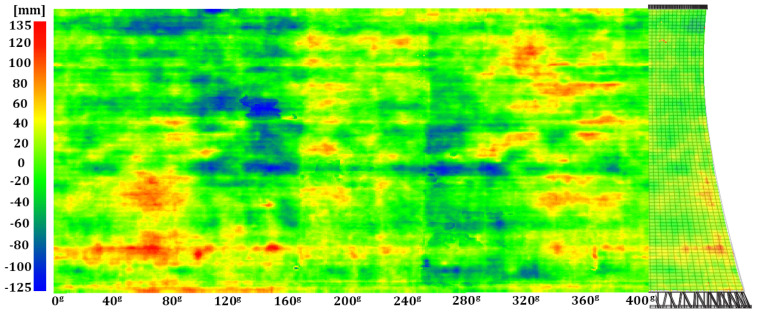
Map of geometric imperfections in the hyperboloid shell: series I. Horizontal: shell development azimuth; vertical: shell height.

**Figure 11:**
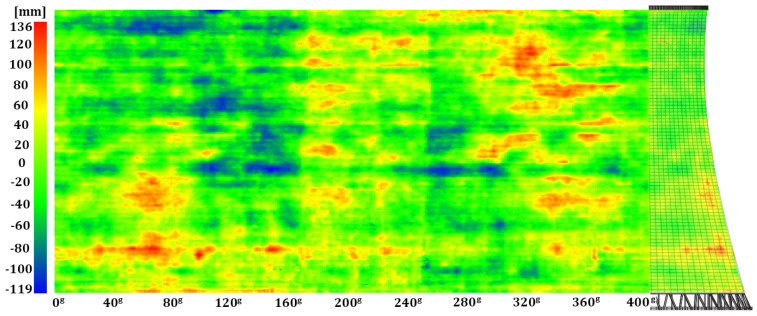
Map of geometric imperfections in the hyperboloid shell: series II. Horizontal: shell development azimuth; vertical: shell height.

**Figure 12:**
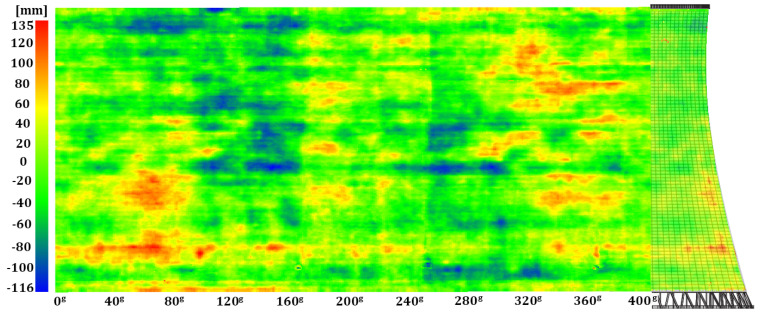
Map of geometric imperfections in the hyperboloid shell: series III. Horizontal: shell development azimuth; vertical: shell height.

Geometric imperfections in the hyperboloid shell were identified by comparing a set of observations describing the internal surface with the model hyperboloid. Thus, identified radial deformations (differences between the measured and theoretical radii) were assigned to each point in the cloud as a specific SF value for the hypsometric visualisation of the results. The imperfections in the reinforced-concrete structure were represented in a three-dimensional space (point clouds) and as a two-dimensional map, which was a metric projection of the scalar-field points onto a side of a cylinder with the base identical to the lower edge of the tower shell developed along it onto a plane (Figure 10, Figure 11 and Figure 12).

We juxtaposed the results of the periodic geometry analysis of the hyperboloid shell with the information on the structure’s geometry found in the company archives. According to the 1990–1991 overhaul documentation, the structure is a hyperboloid of revolution built with panel forms and has a permanent construction defect: local imperfections introduced between construction cycles. The maps generated from the data of the three measurement series (Figure 10, Figure 11 and Figure 12) unambiguously confirmed deformations in critical areas of the structure (especially between the first and second construction cycles) of −0.125 m to +0.136 m.

**Figure 13:**
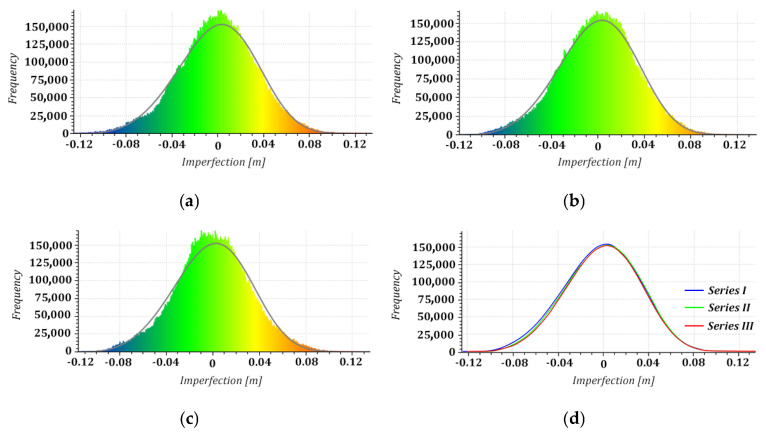
Imperfection histograms with Weibull distribution density curves: (**a**) series I; (**b**) series II; (**c**) series III; (**d**) comparison of Weibull curve plots.

The conformity of the distributions identified for the three measurement series of shape deviations demonstrated a constant geometric state of the structure. Furthermore, we demonstrated no advancing deformation over the two-year research period from histograms with Weibull distribution density curves for each series (Figure 13). The presumed conformity of the imperfection distributions from a visual comparison of the imperfection maps and Weibull curves (Figure 13d) was confirmed with a statistical analysis. Considering the limited computational capacity that could not accommodate all observations, we performed the statistical analysis on representative samples (subsets of 500 imperfections for each series at the nodes of 20 × 25 homothetic grids). The normal distribution of the analysed subsets (samples) was confirmed with a Shapiro–Wilk test at a five per cent significance level (series I: W = 0.996205, *p* = 0.28 > 0.05; series II: W = 0.996568, *p* = 0.37 > 0.05; series III: W = 0.996847, *p* = 0.44 > 0.05). We decided to consider tests of significance of the identified differences in parameter values with a one-way analysis of variance as the foundation for assessing the homogeneity of the results of the three measurement series under relatively constant instrument, team, and ambient conditions. The employed single-factor repeated-measures ANOVA allowed us to simultaneously compare geometric imperfections identified in the three measurement series (assuming the mutual close proximity of variances in differences between all pairs of measurements confirmed with Mauchly’s sphericity test). The hypothesis of homogeneity of the three sets of imperfections was verified at a five per cent significance level (F = 0.02968; *p* = 0.970.05) with no statistically significant differences between the analysed samples resulting from the processing of point clouds from three measurement series. These results were confirmed with a multiple-comparison procedure (post hoc test) to test the differences between each pair of imperfections from the TLS data from the consecutive measurement series. The values of test probabilities (*p* = 0.993, *p* = 0.990, and *p* = 0.968) determined with Tukey’s honest significance test reflecting comparisons between series I–II, I–III, and II–III demonstrated no statistically significant differences between the investigated sets of imperfections. The consistency of the results of the periodic analyses of the shell geometry with the TLS data seemed to confirm the reliability of the proposed solutions and their suitability for regular investigations of the structure’s condition. The results also demonstrated no advancing deformation of the hyperboloid shell, which is vital for its further use.

## 4. Conclusions and Future Work

This article proposes a strategy for using terrestrial laser scanning to investigate the geometry of hyperboloid cooling towers to identify their actual geometric parameters (useful for such purposes as determining their cooling capacity [4]), identify, locate, and estimate the quantity of its geometric imperfections that pose an actual threat to its stability, and ascertain no advancing deformations occur in the shell to secure future service. The solution proposes a reliable identification of geometric imperfections (as the critical drivers of safety risks of thin-shell structures) caused by advancing structural deformations, unrepaired construction defects, or improper reinforcement. In our research, we also included other geometric parameters that are often overlooked and yet affect analytical performance. We also described the object’s suitability for continued service: deviation from plumb and actual shell thickness.

Our empirical tests aimed at verifying the procedures for using information from point clouds facilitated a complete assessment of the geometric condition of the hyperboloid structure, guaranteeing periodic compatibility of reports. Having analysed horizontal cross-sections of the point clouds and the positions of their origins, we determined the permanent deviation of the structure’s axis from plumb (up to 10 mm along the *x*-axis and 52 mm along the *y*-axis) and detected the first signs of shell ovalisation (from the bimodality of the distribution of the roundness deviation of the points in the cross-sections), which urge the administrators to resume foundation settlement monitoring neglected for years. The determination of the distances between the internal and external point clouds with M3C2 allowed us to analyse in detail the distribution of the shell thickness, including its variation due to repairs and reinforcement. We successfully used the identified parameters of the individual thickness distribution zones to reduce the coordinates of the points representing the external surface to their counterpart coordinates of the internal surface, which has to be consistent with a one-sheet hyperboloid. The cloud points reduced to the internal surface facilitated a repeated estimation of the parameters of the structure’s theoretical model (consistent with the data in design documents), which is necessary when design documents are unavailable or unreliable. The analysis of geometric imperfections (the radial distances of the data reduced to the model hyperboloid) based on the observations of the internal surface demonstrated construction errors in the form of deviations from the design geometry across construction cycles ranging from −0.125 m to +0.136 m. The statistically confirmed consistency of imperfections found in consecutive measurement series (indicating consistency across periodic TLS analyses of the geometry of the cooling tower shell) guaranteed the exclusion of any advancing deformations in the shell, which is critical for future service.

The priority in future work will be to conduct case studies illustrating practical applications of the proposed diagnostics concept for assessing large-geometry objects because of the unquestionable difference between the test object and dimensions of modern hyperboloid structures. The primary focus will be to analyse the usefulness of the latest models of terrestrial laser scanners. Other cases when further research is necessary include objects exhibiting significant imperfections or data discontinuity that call for analysis of whether or not to replace the least-squares method (the foundation of the proposed procedures for investigating the geometry of hyperboloid structures) with robust estimation methods.

Another planned path of further research is to employ the proposed solutions to diagnose thin-shell structures and identify the necessary repairs to improve their load capacity and stop imperfections from propagating. In this regard, the proposed concept that can verify information in design documents and reliably analyse the shape and thickness of a hyperboloid shell could ensure an accurate assessment of changes in the parameters of the structure resulting from repairs (contributing to knowledge-based repair cost management and cost verification). The proposed solution may be a valuable tool for the periodic diagnostic monitoring of repairs to verify the effectiveness and lifetime of the improvements, providing a basis for controlling the conformity of repairs and reinforcement with the planned scope of works and cost benefits.

Our next research focus is to analyse the suitability of terrestrial laser scanning to build a diagnostic model of a cooling tower that would accurately imitate the structure’s behaviour under actual service conditions. Numeric simulations that take into consideration the basic loads and materials and geometric non-linearity of the hyperboloid shell will be implemented to account for the nature of structural deformations and damage. They will be considered the foundation for determining the appropriate extent of structural interventions. We further intend to consider the possibility of forecasting the future growth of defects in operational structures as the core of a system for strategic service cost management for hyperboloid cooling towers.

## 5. Patents

The adapters for stabilising the reference spheres on the ground and railings are protected by the Patent Office of the Republic of Poland under utility model nos: 070271, 070272, 071959, and 071960. Inventor: Maria Makuch.

## Figures and Tables

**Table 1:** Summary of results of approximations for theoretical model of structure.

Series I			Series II			Series III		
a [m]	c [m]	$z_{o}$ [m]	a [m]	c [m]	$z_{o}$ [m]	a [m]	c [m]	$z_{o}$ [m]
12.7329	37.8376	51.5462	12.7331	37.8364	51.5459	12.7324	37.8373	51.5470
$\sigma_{o}$ = 0.1252 m			$\sigma_{o}$ = 0.1239 m			$\sigma_{o}$ = 0.1247 m		

**Table 2:** Parameters of structure’s theoretical models.

Identified Parameters	Series I	Series II	Series III	Mean	Method
Radius at the level of the bottom ring	21.5177 m	21.5184 m	21.5172 m	21.5178 m	21.52 m
Throat radius	12.7329 m	12.7331 m	12.7324 m	12.7328 m	12.75 m
Radius at the level of the top ring	13.4914 m	13.4917 m	13.4908 m	13.4913 m	13.52 m

## Data Availability

Data available on request from the corresponding author due to restrictions (the test cooling tower is located in a closed area).

## References

[1] Harte R., Krätzig W.B. (2002). Large-scale cooling towers as part of an efficient and cleaner energy generating technology. Thin Walled Struct..

[2] Wenzel P.M., Mühlen M., Radgen P. (2023). Free Cooling for Saving Energy: Technical Market Analysis of Dry, Wet, and Hybrid Cooling Based on Manufacturer Data. Energies.

[3] Táboas F., Vázquez F. (2021). Pressure Drops and Energy Consumption Model of Low-Scale Closed Circuit Cooling Towers. Processes.

[4] Bernardello R.A., Borin P. (2022). Form follows function in a hyperboloidical cooling Tower. Nexus Netw. J..

[5] Bamu P.C., Zingoni A. (2005). Damage, deterioration and the long-term structural performance of cooling-tower shells: A survey of developments over the past 50 years. Eng. Struct..

[6] Asadzadeh E., Alam M. (2014). A Survey on Hyperbolic Cooling Towers. Int. J. Civ. Struct. Constr. Archit. Eng..

[7] Lingaraju M.K.C., Girisha S.K., Channabasappa S.B., Karigowda M.A. (2021). Study on Dynamic Behavior of Natural Draft Cooling Tower Considering the Effect of Soil-Structure Interaction. Civ. Environ. Eng. Rep..

[8] Zahradník D. (2023). Cooling tower measurement by laser scanner and close-range photogrammetry. AIP Conf. Proc..

[9] Hojdys Ł., Krajewski P., Seręga S., Płachecki M. (2012). Stan techniczny powłoki żelbetowej hiperboloidalnej chłodni kominowej z dużymi imperfekcjami po 35 latach użytkowania [The technical condition of the reinforced concrete shell of a hyperboloid cooling tower with significant imperfections after 35 years of use]. Przegląd Bud..

[10] Makuch M. (2018). Application of Terrestrial Laser Scanning in the Process of Modernization of Hyperboloid Cooling Towers. Ph.D. Thesis.

[11] Kocierz R., Rebisz M., Łukasz O. (2018). Measurement point density and measurement methods in determining the geometric imperfections of shell surfaces. Rep. Geod. Geoinform..

[12] Wenjie L., Shitang K., Yang J., Wu H., Wang F., Han G. (2022). Wind-induced collapse mechanism and failure criteria of super-large cooling tower based on layered shell element model. J. Wind Eng. Ind. Aerodyn..

[13] Kamiński M. (2010). Wybrane problemy diagnostyki i oceny stanu technicznego chłodni kominowych [Selected problems of diagnostics and assessment of the technical condition of cooling towers]. Przegląd Bud..

[14] Ejsymont M., Prusiel J.A. (2023). Analiza dynamiczna chłodni kominowych [Dynamic analysis of cooling towers]. Przegląd Bud..

[15] Głowacki T., Muszyński Z. (2018). Analysis of cooling tower’s geometry by means of geodetic and thermovision method. IOP Conf. Ser. Mater. Sci. Eng..

[16] Muszyński Z. (2013). Zastosowanie metody Hampela do aproksymacji modelu teoretycznego chłodni kominowej w podejściu dwuwymiarowym [Application of the Hampel’s method to approximate a theoretical model of the cooling tower in the two-dimensional approach]. Arch. Fotogram. Kartogr. I Teledetekcji.

[17] Zdanowicz K. (2011). Geodezyjny monitoring deformacji powierzchni hiperboloidalnych chłodni kominowych [Geodetic monitoring of surface deformation of hyperboloid cooling towers]. Czas. Tech. Bud..

[18] Gould P.L., Krätzig W.B., Wai-Fah C. (1999). Cooling Tower Structures, Structural Engineering Handbook.

[19] Ioannidis C., Valani A., Georgopoulos A., Tsiligiris E. 3D model generation for deformation analysis using laser scanning data of a cooling tower. Proceedings of the 3rd IAG 12th FIG Symposium on Deformation Measurements.

[20] Camp G., Carreaud P., Lançon H. (2013). Large Structures: Which Solutions for Health Monitoring?. Int. Arch. Photogramm. Remote Sens. Spat. Inf. Sci..

[21] Piot S., Lancon H. New Tools for the Monitoring of Cooling Towers. Proceedings of the 6th European Workshop on Strutural Health Montoring.

[22] Antoniszyn K., Hawro L., Konderla P., Kutyłowski R. (2016). Wybrane problemy procesów modernizacji i remontów chłodni kominowych. [Selected issues with upgrading and repairing cooling towers]. Mater. Bud..

[23] Tan G. (2009). Application of 3D Laser Scanner in the Deformation Monitoring of Cooling Towers in Power Plant. Electr. Power Surv. Des..

[24] Holst C., Kuhlmann H. Challenges and present fields of action at laser scanner based deformation analyses. Proceedings of the 3rd Joint International Symposium on Deformation Monitoring (JISDM).

[25] Mukupa W., Roberts G.W., Hancock C.M., Al-Manasir K. (2017). A review of the use of terrestrial laser scanning application for change detection and deformation monitoring of structures. Surv. Rev..

[26] Gordon S.J., Lichti D.D. (2007). Modelling terrestrial laser scanner data for precise structural deformation measurement. J. Surv. Eng..

[27] Vosselman G., Mass H.G. (2010). Airborne and Terrestrial Laser Scanning.

[28] Vezocnik R., Ambrozic T., Sterle O., Bilban G., Pfeifer N., Stopar B. (2009). Use of terrestrial laser scanning technology for long term high precision deformation monitoring. Sensors.

[29] Lindenbergh R., Uchanski L., Bucksch A., Van Gosliga R. (2009). Structural monitoring of tunnels using terrestrial laser scanning. Rep. Geod..

[30] Lovas T., Barsi A., Detrekoi A., Dunai L., Csak Z., Polgar A., Berenyi A., Kibedy Z., Szocs K. (2008). Terrestrial laser scanning in deformation measurements of structures. Int. Arch. Photogramm. Remote Sens. Spat. Inf. Sci..

[31] Gordon S.J., Lichti D.D., Stewart M., Franke J. Structural deformation measurements using terrestrial laser scanners. Proceedings of the 11th FIG Symposium on Deformation Measurements.

[32] Monserrat O., Crosetto M. (2008). Deformation measurement using terrestrial laser scanning data and least squares 3D surface matching. ISPRS J. Photogramm. Remote Sens..

[33] Zhao X., Kargoll B., Omidalizarandi M., Xu X., Alkhatib H. (2018). Model Selection for Parametric Surfaces Approximating 3D Point Clouds for Deformation Analysis. Remote Sens..

[34] Paffenholz J.A., Lu C. 3D point cloud based spatio-temporal monitoring of artificial and natural objects. Proceedings of the FIG Working Week Smart, Surveyors for Land and Water Management.

[35] Han J., Guo J., Jiang Y. (2013). Monitoring tunnel profile by means of multi-epoch dispersed 3-D LIDAR point clouds. Tunn. Undergr. Space Technol..

[36] Yi C., Lu D., Xie Q., Xu J., Wang J. (2020). Tunnel Deformation Inspection via Global Spatial Axis Extraction from 3D Raw Point Cloud. Sensors.

[37] Wróblewski A., Wodecki J., Trybała P., Zimroz R. (2022). A Method for Large Underground Structures Geometry Evaluation Based on Multivariate Parameterization and Multidimensional Analysis of Point Cloud Data. Energies.

[38] Mosalam K.M., Shakhzod M., Park S.T. (2014). Applications of laser scanning to structures in laboratory tests and field surveys. Struct. Control Health Monit..

[39] Xie X., Lu X. (2017). Development of a 3D modeling algorithm for tunnel deformation monitoring based on terrestrial laser scanning. Undergr. Space.

[40] Owerko T., Kuras P., Ortyl Ł., Kocierz R. (2012). Wykorzystanie skaningu laserowego do wyznaczania deformacji stalowych wież telekomunikacyjnych [The use of laser scanning to determine the deformation of steel telecommunications towers]. Pomiary Autom. Kontrola.

[41] Teza G., Pesci A. (2013). Geometric characterization of a cylinder-shaped structure from laser scanner data: Development of an analysis tool and its use on a leaning bell tower. J. Cult. Herit..

[42] Janus J., Ostrogórski P. (2022). Underground Mine Tunnel Modelling Using Laser Scan Data in Relation to Manual Geometry Measurements. Energies.

[43] Lu Z., Gong H., Jin Q., Hu Q., Wang S. (2022). A Transmission Tower Tilt State Assessment Approach Based on Dense Point Cloud from UAV-Based LiDAR. Remote Sens..

[44] Schneider D. Terrestrial laser scanning for area based deformation analysis of towers and water dams. Proceedings of the 3rd IAG/12th FIG Symposium.

[45] Głowacki T. (2022). Monitoring the Geometry of Tall Objects in Energy Industry. Energies.

[46] Gawałkiewicz R. (2007). Przykład skanowania laserowego w monitoringu obiektów powłokowych [An example of laser scanning in monitoring shell objects]. Geomat. Environ. Eng..

[47] Beshr A.A.A., Basha A.M., El-Madany S.A., El-Azeem F.A. (2023). Deformation of High Rise Cooling Tower through Projection of Coordinates Resulted from Terrestrial Laser Scanner Observations onto a Vertical Plane. ISPRS Int. J. Geo Inf..

[48] Kocierz R., Ortyl Ł., Kuras P., Owerko T., Kędzierski M. (2016). Geodezyjne metody pomiarowe w diagnostyce obiektów budownictwa energetycznego [Geodetic measurement methods in the diagnostics of energy construction facilities]. Mater. Bud..

[49] Noakowski P. (2010). Ocena wytężenia chłodni kominowej wskutek nierównomiernych osiadań jej fundamentów [Assessment of the cooling tower stress due to uneven settlement of its foundations]. Przegląd Bud..

[50] Besl P.J., McKay N.D. (1992). A method for registration of 3-d shapes. IEEE Trans. Pattern Anal. Mach. Intell..

[51] Gruen A., Akca D. (2005). Least sequares 3D surface and curve matching. ISPRS J. Photogramm. Remote Sens..

[52] Girardeau-Montaut D., Roux M., Marc R., Thibault G. (2005). Change detection on points cloud data acquired with a ground laser scanner. Int. Arch. Photogramm. Remote Sens. Spat. Inf. Sci..

[53] Lague D., Brodu N., Leroux J. (2013). Accurate 3D comparison of complex topography with terrestrial laser scanner: Application to the Rangitikei canyon (N–Z). ISPRS J. Photogramm. Remote Sens..

[54] Lindenbergh R., Pfeifer N. A statistical deformation analysis of two epochs of terrestrial laser data of a lock. Proceedings of the Optical 3D Measurement Techniques VII/2.

[55] Antova G. Terrestrial Laser Scanning for Dam Deformation Monitoring—Case Study. Proceedings of the FIG Working Week from the Wisdom of the Ages to the Challenges of the Modern World.

[56] Kromer R.A., Abellán A., Hutchinson D.J., Lato M., Edwards T., Jaboyedoff M. (2015). A 4D Filtering and Calibration Technique for Small-Scale Point Cloud Change Detection with a Terrestrial Laser Scanner. Remote Sens..

[57] Law D.W., Silcock D., Holden L. (2018). Terrestrial laser scanner assessment of deteriorating concrete structures. Struct. Control Health Monit..

[58] Yang Y., Schwieger V. (2023). Patch-based M3C2: Towards lower-uncertainty and higher-resolution deformation analysis of 3D point clouds. Int. J. Appl. Earth Obs. Geoinf..

[59] Walton G., Delaloye D., Diederichs M.S. (2014). Development of an elliptical fitting algorithm to improve change detection capabilities with applications for deformation monitoring in circular tunnels and shafts. Tunn. Undergr. Space Technol..

[60] Lindenbergh R., Pietrzyk P. (2015). Change detection and deformation analysis using static and mobile laser scanning. Appl. Geomat..

[61] Van Gosliga R., Lindenbergh R., Pfeifer N. (2006). Deformation analysis of a bored tunnel by means of terrestrial laser scanning. Int. Arch. Photogramm. Remote Sens. Spat. Inf. Sci..

[62] Tsakiri M., Lichti D., Pfeifer N. Terrestrial laser scanning for deformation monitoring. Proceedings of the 3rd IAG/12th FIG Symposium.

[63] Barnhart T.B., Crosby B.T. (2013). Comparing Two Methods of Surface Change Detection on an Evolving Thermokarst Using High-Temporal-Frequency Terrestrial Laser Scanning, Selawik River, Alaska. Remote Sens..

[64] Zogg H.M., Ingensand H. (2008). Terrestrial laser scanning for deformation monitoring—Load tests on the Felsenau viaduct. Int. Arch. Photogramm. Remote Sens. Spat. Inf. Sci..

[65] Zahs V., Hämmerle M., Anders K., Hecht S., Sailer R., Rutzinger M., Williams J.G., Höfle B. (2019). Multi-temporal 3D point cloud-based quantification and analysis of geomorphological activity at an alpine rock glacier using airborne and terrestrial LiDAR. Permafr. Periglac. Process..

[66] DiFrancesco P.M., Bonneau D., Hutchinson D.J. (2020). The Implications of M3C2 Projection Diameter on 3D Semi-Automated Rockfall Extraction from Sequential Terrestrial Laser Scanning Point Clouds. Remote Sens..

[67] Winiwarter L., Anders K., Höfle B. (2021). M3C2-EP: Pushing the limits of 3D topographic point cloud change detection by error propagation. ISPRS J. Photogram. Remote Sens..

[68] Holst C., Klingbeil L., Esser F., Kuhlmann H. Using point cloud comparisons for revealing deformations of natural and artificial objects. Proceedings of the INGEO 2017, 7th International Conference on Engineering Surveying.

[69] Paffenholz J.A., Huge J., Stenz U. (2018). Integration of Laser Tracking and Laser Scanning for Optimal Detection of Load Induced Arch Displacement. Allg. Vermess. Nachrichten.

[70] Abdelazeem M., Elamin A., Afifi A., El-Rabbany A. (2021). Multi-sensor point cloud data fusion for precise 3D mapping. Egypt. J. Remote Sens. Space Sci..

[71] Pu X., Gan S., Yuan X., Li R. (2022). Feature Analysis of Scanning Point Cloud of Structure and Research on Hole Repair Technology Considering Space-Ground Multi-Source 3D Data Acquisition. Sensors.

[72] Morgan J., Ferrell J., Schultz C. (2023). Jasiak. Photogrammetric Point-Cloud Replicability When Documenting Forensic Archaeological Scenes under Variable Lighting Conditions. Tech. Notes.

[73] Zahs V., Winiwarter L., Anders K., Williams J.G., Rutzinger M., Höfle B. (2022). Correspondence-driven plane-based M3C2 for lower uncertainty in 3D topographic change quantification. ISPRS J. Photogram. Remote Sens..

[74] González-Aguilera D., Gómez-Lahoz J., Sánchez J. (2008). A New Approach for Structural Monitoring of Large Dams with a Three-Dimensional Laser Scanner. Sensors.

[75] Takhirov S., Rakhmonov B. Structural Health Monitoring of the Juma Mosque in Itchan Kala in Khiva (Uzbekistan): Laser Scanning Combined with Numerical Modelling. Proceedings of the 12th International Conference on Structural Analysis of Historical Constructions SAHC.

[76] Harmening C., Hobmaier C., Neuner H. (2021). Laser Scanner–Based Deformation Analysis Using Approximating B-Spline Surfaces. Remote Sens..

[77] Guidi G., Malik U.S., Manes A., Cardamone S., Fossati M., Lazzari C., Volpato C., Giglio M. (2020). Laser Scanner-Based 3D Digitization for the Reflective Shape Measurement of a Parabolic Trough Collector. Energies.

[78] Glowacki T., Grzempowski P., Sudol E., Wajs J., Zajac M. (2016). The assessment of the application of terrestrial Laser scanning for measuring the geometrics of cooling tower. Geomat. Landmanag. Landsc..

[79] Kwinta A., Bac-Bronowicz J. (2021). 2021. Analysis of hyperboloid cooling tower projection on 2D shape. Geomat. Landmanag. Landsc..

[80] Mohamed A., Wilkinson B. (2009). Direct Georeferencing of Stationary LiDAR. Remote Sens..

[81] Paffenholz J.A., Kutterer H. Direct georeferencing of static terrestrial laser scans. Proceedings of the FIG Working Week.

[82] Becerik-Gerber B., Farrokh J., Geoffrey K., Gulben C. (2011). Assessment of target types and layouts in 3D laser scanning for registration accuracy. Autom. Constr..

[83] Du S.Y., Zheng N.N., Xiong L., Ying S.H., Xue J.R. (2010). Scaling iterative closest point algorithm for registration of m–D point sets. J. Vis. Commun. Image Represent..

[84] Elseberg J., Borrmann D., Nüchter A. (2013). One billion points in the cloud—An octree for efficient processing of 3D laser scans. ISPRS J. Photogramm. Remote Sens..

[85] Zwillinger D., Zwillinger D. (2002). CRC Standard Mathematical Tables and Formulae.

